# Breakthrough infections in MPN-COVID vaccinated patients

**DOI:** 10.1038/s41408-022-00749-8

**Published:** 2022-11-15

**Authors:** Tiziano Barbui, Alessandra Carobbio, Arianna Ghirardi, Alessandra Iurlo, Valerio De Stefano, Marta Anna Sobas, Elisa Rumi, Elena Maria Elli, Francesca Lunghi, Mercedes Gasior Kabat, Beatriz Cuevas, Paola Guglielmelli, Massimiliano Bonifacio, Monia Marchetti, Alberto Alvarez-Larran, Laura Fox, Marta Bellini, Rosa Daffini, Giulia Benevolo, Gonzalo Carreno-Tarragona, Andrea Patriarca, Haifa Kathrin Al-Ali, Maria Marcio Miguel Andrade-Campos, Francesca Palandri, Claire Harrison, Maria Angeles Foncillas, Santiago Osorio, Steffen Koschmieder, Elena Magro Mazo, Jean-Jacques Kiladjian, Estefanía Bolaños Calderón, Florian H. Heidel, Keina Quiroz Cervantes, Martin Griesshammer, Valentin Garcia-Gutierrez, Alberto Marin Sanchez, Juan Carlos Hernandez-Boluda, Emma Lopez Abadia, Giuseppe Carli, Miguel Sagues Serrano, Rajko Kusec, Blanca Xicoy Cirici, Margarita Guenova, Begona Navas Elorza, Anna Angona, Edyta Cichocka, Anna Kulikowska de Nałęcz, Daniele Cattaneo, Cristina Bucelli, Silvia Betti, Oscar Borsani, Fabrizio Cavalca, Sara Carbonell, Natalia Curto-Garcia, Lina Benajiba, Alessandro Rambaldi, Alessandro Maria Vannucchi

**Affiliations:** 1 FROM Research Foundation, Papa Giovanni XXIII Hospital, Bergamo, Italy; 2 Hematology Division, Foundation IRCCS Ca’ Granda Ospedale Maggiore Policlinico, Milan, Italy; 3 Dipartimento di Scienze Radiologiche ed Ematologiche, Sezione di Ematologia, Università Cattolica del Sacro Cuore – Policlinico Universitario Agostino Gemelli IRCCS, Rome, Italy; 4 Department of Hematology, Blood Neoplasms and Bone Marrow Transplantation, Wroclaw Medical University, Wrocław, Poland; 5 Department of molecular medicine, University of Pavia, Pavia, Italy; 6 Hematology Division and Bone Marrow Transplant Unit, San Gerardo Hospital, ASST Monza, Monza, Italy; 7 IRCCS Ospedale San Raffaele, Milano, Italy; 8 Hospital Universitario La Paz, Madrid, Spain; 9 Hospital Universitario de Burgos, Burgos, Spain; 10 Center Research and Innovation of Myeloproliferative Neoplasms (CRIMM), Department of Experimental and Clinical Medicine, Azienda Ospedaliera Universitaria Careggi, University of Florence, Florence, Italy; 11 Department of Medicine, University of Verona, Azienda Ospedaliera Universitaria Integrata, Verona, Italy; 12 AOU SS.Antonio e Biagio e C.Arrigo, Alessandria, Italy; 13 Hospital Clinic de Barcelona, Barcelona, Spain; 14 Department of Hematology, Vall d’Hebron Institute of Oncology (VHIO), Vall d’Hebron Hospital Universitari, Vall d’Hebron Barcelona Hospital Campus, C/Natzaret, 115-117, 08035 Barcelona, Spain; 15 ASST Papa Giovanni XXIII, Bergamo, Italy; 16 ASST-Spedali Civili, Brescia, Italy; 17 AOU Città della Salute e della Scienza di Torino, Torino, Italy; 18 Hospital Universitario 12 de Octubre, Madrid, Spain; 19 AOU Maggiore della Carità, Novara, Italy; 20 University Hospital Halle, Department of Hematology/Oncology, 06112 Halle (Saale), Germany; 21 Hospital del Mar, Barcelona, Spain; 22 IRCCS Azienda Ospedaliero-Universitaria di Bologna, Bologna, Italy; 23 Guy’s and St. Thomas’ NHS Foundation Trust, London, United Kingdom; 24 Hospital Universitario Infanta Leonor, Madrid, Spain; 25 Hospital Gregorio Maranon, Madrid, Spain; 26 Department of Hematology, Oncology, Hemostaseology, and Stem Cell Transplantation, Faculty of Medicine, RWTH Aachen University, Aachen, Germany; 27 Hospital Universitario Principe de Asturias, Alcalà de Henares, Madrid, Spain; 28 Hospital Saint-Louis, Paris, France; 29 Hospital Clinico San Carlos, Madrid, Spain; 30 University Medicine Greifswald - Hematology, Oncology, Stem Cell Transplantation and Palliative Care Sauerbruchstraße, 17475 Greifswald, Germany; 31 Hospital Universitario de Mostoles, Madrid, Spain; 32 University Clinic for Hematology, Oncology, Hemostaseology and Palliative Care, Johannes Wesling Medical Center, Minden, Germany; 33 Hospital Ramon y Cajal, IRYCIS, Madrid, Spain; 34 Hospital General Universitario de Albacete, Albacete, Spain; 35 Hospital Clinico Universitario, INCLIVA, Valencia, Spain; 36 Hospital General de Elche, Elche (Alicante), Spain; 37 Ospedale San Bortolo, Vicenza, Italy; 38 ICO L’Hospitalet-Hospital Moises Broggi, Sant Joan Despì, Barcelona, Spain; 39 University Hospital Dubrava-School of Medicine University of Zagreb, Department of haematology, Clinic of internal medicine, Zagreb, Croatia; 40 ICO Hospital Germans Trias i Pujol, Badalona (Barcelona), Josep Carreras Leukemia Research Institute, Universitat Autònoma de Barcelona, Barcelona, Spain; 41 National Specialised Hospital for Active Treatment of Hematological Diseases, Sofia, Bulgaria; 42 Hospital Moncloa, Madrid, Spain; 43 ICO Girona Hospital Josep Trueta, Girona, Spain; 44 Department of Hematology and Bone Marrow Transplantation, Nicolaus Copernicus Hospital, Torun, Poland; 45 Department of Hematology and Internal Diseases, State Hospital, Opole, Poland; 46 Università degli Studi di Milano, Milano, Italy

**Keywords:** Myeloproliferative disease, Myeloproliferative disease

The first wave of COVID-19 in patients with myeloproliferative neoplasms (MPN) including polycythemia vera (PV), essential thrombocythemia (ET), prefibrotic myelofibrosis (pre-PMF) and overt myelofibrosis (MF), was characterized by severe illness, high hospitalization rate, and excess of mortality [1, 2]. Deaths accounted for 28.5% of patients [1], an estimate 3-4-fold higher than in general population [3], and progressively decreased thereafter [4]. This improvement was likely related to better critical care management, less virulence of circulating variants of concern (VOCs), younger patient’s age and vaccinations. The effectiveness of vaccines to protect against disease severity, hospitalization and death has been consistently demonstrated in the general population; [5] conversely, very limited information has been provided so far in rare diseases such as MPNs [6, 7] in which vaccines elicit poor neutralizing antibody titers, particularly in MF on ruxolitinib active treatment [8, 9].

We investigated 3 cohorts of COVID-MPN patients, observed since the beginning of the SARS-CoV2, from February 2020 to June 2022. In the European MPN-COVID registry, promoted by the LeukemiaNet (ELN) (clinicaltrials.gov: NCT04385160), 863 MPN patients with COVID-19 have been enrolled, and in 649 of them, information on the vaccination status was provided. diagnosis of covid-19 required a positive real-time reverse transcriptase polymerase chain reaction from nasal swab and symptoms highly suggestive for sars-cov-2 infection. according to the most prevalent circulating vocs in europe at that time, 4 waves were identified: 1st, February to June 2020 (wild-type original variant) 2nd, July 2020–June 2021 (alpha/beta/gamma); 3rd, July 2021–December 2021 (delta); and wave 4th, January to June 2022 (Omicron). The severity of COVID-19 was categorized as asymptomatic, mild, moderate or severe/critical according to the NIH COVID-19 Treatment Guidelines [10].

## Infections in vaccinated patients with previous COVID-19 (hybrid vaccination)

Of 418 patients with prior COVID-19, 287 were vaccinated and 131, at the moment of the present analysis, were not. Almost all vaccinated cases (98%) had prior COVID-19 during the first and second pandemic wave. In unvaccinated cases, prior COVID-19 occurred frequently during the last 2 waves, including Delta and Omicron VOCs (26%) (Table 1). Of note, unvaccinated cases experienced the first episode of COVID-19 less severe than vaccinated ones (i.e., asymptomatic in 7.3% vs. 2.6%, respectively, *p* = 0.003) and were younger (median age 59 years vs. 62 years, respectively, *p* = 0.016). The proportion of PV, ET, pre-PMF was similar in the two groups while the proportion of MF patients was lower in the vaccinated than unvaccinated group (19% vs 28%) (*p* = 0.024). No difference of driver mutations frequency and splenomegaly among the MPN phenotypes was found.

**Table 1:** Characteristics of patients with previous COVID-19 infection according to vaccination status.

	Total pts with previous infection	UNVACCINATED	VACCINATED	*p*
	*N* = 418	*N* = 131	*N* = 287	
Male gender	189/418 (45.2%)	51/131 (38.9%)	138/287 (48.1%)	0.081
Age	61.3 (53.0–71.7)	59.3 (47.9–71.9)	61.8 (54.6–71.7)	0.016
> 70	124/417 (29.7%)	36/131 (27.5%)	88/286 (30.8%)	0.50
BMI	24.0 (21.3–26.5)	24.4 (21.7–26.1)	23.9 (21.3–26.5)	0.89
MPN diagnosis				
ET	156/418 (37.3%)	41/131 (31.3%)	115/287 (40.1%)	0.085
PV	133/418 (31.8%)	41/131 (31.3%)	92/287 (32.1%)	0.88
MF	90/418 (21.5%)	37/131 (28.2%)	53/287 (18.5%)	0.024
Early/pre-PMF	39/418 (9.3%)	12/131 (9.2%)	27/287 (9.4%)	0.94
Previous thrombosis	81/415 (19.5%)	24/129 (18.6%)	57/286 (19.9%)	0.75
Mutational status				
*JAK2 V617F*	303/408 (74.3%)	93/127 (73.2%)	210/281 (74.7%)	0.75
*CALR*	64/231 (27.7%)	19/89 (21.3%)	45/142 (31.7%)	0.087
* MPL*	10/220 (4.5%)	5/88 (5.7%)	5/132 (3.8%)	0.53
* JAK2 EXON12*	1/126 (0.8%)	1/55 (1.8%)	0/71 (0.0%)	0.44
Spleen palpable				
No	270/386 (69.9%)	88/125 (70.4%)	182/261 (69.7%)	
Yes	111/386 (28.8%)	35/125 (28.0%)	76/261 (29.1%)	0.87
Previously splenectomized	5/386 (1.3%)	2/125 (1.6%)	3/261 (1.1%)	
Size below costal margin (cm)	4.0 (2.0–6.0)	4.5 (2.0–6.5)	3.0 (2.0–6.0)	0.30
MPN treatment post-COVID				
Phlebotomy	76/410 (18.5%)	24/130 (18.5%)	52/280 (18.6%)	0.98
Cytoreduction	289/418 (69.1%)	83/131 (63.4%)	206/287 (71.8%)	0.084
HU	196/412 (47.6%)	54/130 (41.5%)	142/282 (50.4%)	0.096
Anagrelide	13/412 (3.2%)	1/130 (0.8%)	12/282 (4.3%)	0.071
Interferon	12/412 (2.9%)	3/130 (2.3%)	9/282 (3.2%)	0.76
Ruxolitinib	63/412 (15.3%)	20/130 (15.4%)	43/282 (15.2%)	0.97
Antiplatelets post-COVID	250/410 (61.0%)	75/129 (58.1%)	175/281 (62.3%)	0.43
ASA	233/412 (56.6%)	73/130 (56.2%)	160/282 (56.7%)	0.91
Anticoagulants	95/409 (23.2%)	32/128 (25.0%)	63/281 (22.4%)	0.57
Characteristics of COVID-19				
Wave				
1 (Wild-type)	94/418 (22.5%)	14/131 (10.7%)	80/287 (27.9%)	<0.001
2 (Alpha, Beta, Gamma)	284/418 (67.9%)	83/131 (63.4%)	201/287 (70.0%)	0.175
3 (Delta)	19/418 (4.5%)	17/131 (13.0%)	2/287 (0.7%)	<0.001
4 (Omicron)	21/418 (5.0%)	17/131 (13.0%)	4/287 (1.4%)	<0.001
COVID-19 severity^a^				
Asymptomatic infection	16/389 (4.1%)	9/123 (7.3%)	7/266 (2.6%)	0.003
Mild Illness	286/389 (73.5%)	84/123 (68.3%)	202/266 (75.9%)	0.11
Moderate Illness	25/389 (6.4%)	7/123 (5.7%)	18/266 (6.8%)	0.69
Severe/critical Illness	62/389 (15.9%)	23/123 (18.7%)	39/266 (14.7%)	0.31
Vaccine information				
Months since prior infection	**–**	**–**	5.9 (4.3–11.2)	**–**
No administered doses				
Only 1 dose	–	–	109/287 (38.0%)	–
2 doses	–	–	112/287 (39.0%)	–
3–4 doses	–	–	66/287 (23.0%)	–
Vaccine type				
Pfizer/BioNTech	–	–	204/287 (71.1%)	–
Moderna	–	–	53/287 (18.5%)	–
AstraZeneca	–	–	12/287 (4.2%)	–
Johnson&Johnson	–	–	3/287 (1.0%)	–
NA	–	–	15/287 (5.2%)	–
Reinfections	18 (4.3%)	8 (6.1%)	10 (3.5%)	0.22
Months to reinfection from prior COVID-19	13.1 (8.2–15.0)	7.5 (4.8–13.1)	14.3 (12.9–16.8)	0.016
Months to reinfection from last vaccine dose	**–**	–	3.1 (2.5–7.1)	–
Number of doses				
Only 1 dose	–	–	2/10 (20.0%)	
2 doses	–	–	4/10 (40.0%)	–
3–4 doses	–	–	4/10 (40.0%)	
Wave of reinfection				
3 (Delta)	4/18 (22.2%)	3/8 (37.5%)	1/10 (10.0%)	0.27
4 (Omicron)	14/18 (77.8%)	5/8 (62.5%)	9/10 (90.0%)	
COVID-19 severity^a^				
Asymptomatic infection	2/17 (11.8%)	1/8 (12.5%)	1/9 (11.1%)	0.72
Mild Illness	13/17 (76.5%)	7/8 (87.5%)	6/9 (66.7%)	
Severe/critical Illness	2/17 (11.8%)	0/8 (0.0%)	2/9 (22.2%)	
Patient disposition				
Home-treated	16/18 (88.9%)	8/8 (100.0%)	8/10 (80.0%)	0.48
Hospitalized	2/18 (11.1%)	0/8 (0.0%)	2/10 (20.0%)	
Respiratory support	1/18 (5.6%)	0/8 (0.0%)	1/10 (10.0%)	1.00
ICU	1/18 (5.6%)	0/8 (0.0%)	1/10 (10.0%)	1.00
Symptoms				
Fever	5/18 (27.8%)	3/8 (37.5%)	2/10 (20.0%)	0.61
Cough	7/18 (38.9%)	1/8 (12.5%)	6/10 (60.0%)	0.066
Dyspnea	3/18 (16.7%)	0/8 (0.0%)	3/10 (30.0%)	0.22
Systemic	1/18 (5.6%)	0/8 (0.0%)	1/10 (10.0%)	1.00
Gastrointestinal	1/18 (5.6%)	0/8 (0.0%)	1/10 (10.0%)	1.00
Fatigue	6/18 (33.3%)	2/8 (25.0%)	4/10 (40.0%)	0.64
Outcome reinfections				
Death^b^	1/18 (5.6%)	0/8 (0.0%)	1/10 (10.0%)	1.00

^a^According to the NIH COVID-19 Treatment Guidelines.

^b^The dead patient was male with age > 70 years old, had a MF diagnosis treated with fedratinib (in a clinical trial) and had a severe breakthrough infection in the Omicron period (symptoms: cough, dyspnea, fatigue) after 2 doses of vaccine and needed invasive respiratory support.

Vaccine doses (COVID-19 mRNA vaccine—Pfizer/BioNTech in 71%) were 1–2 and 3–4 in 77 and 23%, respectively. Only 3 patients (1%) received 4 vaccine shots.

Adverse events (AE) attributed to vaccines (fever, headache) occurred in 8 patients (2.8%). No patient reported serious adverse events including thrombosis.

Overall, 18 reinfections were diagnosed; of these, 4 occurred during Delta and 14 during Omicron variants period (Table 1). Eight (6.1%) were recorded in unvaccinated and 10 (3.5%) in vaccinated patients (*p* = 0.22). Therefore, a statistically significant benefit of vaccination in individuals with prior COVID-19 was not apparent; however, these findings were based on a small number of reinfections and need confirmation by prolonging the follow-up in longitudinal studies to assess the durability of the protection in comparable groups [11].

Of note, among patients who developed reinfections, 9/10 (90%) and 5/8 (63%) occurred during Omicron period in vaccinated and unvaccinated groups, respectively. The time interval between the first SARS-Cov-2 infection and the reinfection was double in vaccinated than in unvaccinated patients (14.3 vs. 7.5 months, respectively, *p* = 0.016), suggesting that booster or repeat vaccination is important and the immunity wanes with time. Time from last vaccine shot to reinfection was 3.1 months.

Severity of reinfection was mild in the great majority of both unvaccinated and vaccinated patients (88 and 67%, respectively, *p* = 0.72).

Overall, compared with the normal population, partial and fully hybrid vaccination in our MPN patient cohorts conferred a 10-fold lower protection (3.5%) than that reported in Italy (0.4%) [12] and in 2 US cohorts in New York city (0.4%) [13] and California (0.3%) [13]. Notably, this poor result was limited to myelofibrosis and to exposure to ruxolitinib in which the rate of reinfections was not different from that registered in severely immunocompromised patients [14].

## Breakthrough infections in vaccinated patients without previous Covid-19 infection

Breakthrough infections were reported in vaccinated patients (ET = 89, PV = 75, MF = 54 and pre-PMF = 13) who had no previous history of COVID-19. Of these, 26 (11%) were hospitalized and their characteristics, compared with home-treated cases, are reported in Table 2. Hospitalized patients were more likely to be older (median age 76 years), males (69%), afflicted with MF (39%), and to have had prior exposure to ruxolitinib (42%: 7 MF and 4 PV). Of note, baseline values at COVID-19 diagnosis of C-reactive protein (CRP) and neutrophil to lymphocyte ratio (NLR) were significantly higher in hospitalized than in home-managed cases (CRP = 33.1 mg/dl vs. 2.0 and NLR = 5.9 vs. 3.3, *p* < 0.001). Although some infections occurred in the second wave, corresponding to Alpha/Beta/Gamma VOCs, the majority of breakthrough episodes occurred during Delta and Omicron variant periods (41% and 53%, respectively), and illness severity was mild in the great majority (86%), even though a moderate to severe infection accounted for more than half of hospitalized patients. Five deaths were registered: 3 and 2 in hospitalized and home managed cases, respectively (12% vs. 1%, *p* = 0.011). The number of vaccine doses was the same in the 2 groups, and patients fully vaccinated (3–4 shots) were 42% and 41%, respectively. Mild adverse events (AE) attributed to vaccination were seen in only 3 patients (1.3%).

**Table 2:** Characteristics of vaccine breakthrough infections according to the severity of Covid-19.

	Total pts without previous infection	HOME-TREATED	HOSPITALIZED	*p*
	*N* = 231	*N* = 205	*N* = 26	
Male gender	105/231 (45.5%)	87/205 (42.4%)	18/26 (69.2%)	0.010
Age at COVID diagnosis	60.4 (49.8–73.2)	57.4 (49.2–71.5)	75.5 (67.3–84.3)	<0.001
>70	71/231 (30.7%)	55/205 (26.8%)	16/26 (61.5%)	<0.001
MPN diagnosis				
ET	89/231 (38.5%)	80/205 (39.0%)	9/26 (34.6%)	0.66
PV	75/231 (32.5%)	68/205 (33.2%)	7/26 (26.9%)	0.52
MF	54/231 (23.4%)	44/205 (21.5%)	10/26 (38.5%)	0.054
Early/pre-PMF	13/231 (5.6%)	13/205 (6.3%)	0/26 (0.0%)	0.19
Previous thrombosis	36/231 (15.6%)	30/205 (14.6%)	6/26 (23.1%)	0.26
Mutational status				
*JAK2 V617F*	166/227 (73.1%)	147/203 (72.4%)	19/24 (79.2%)	0.48
*CALR*	34/136 (25.0%)	32/126 (25.4%)	2/10 (20.0%)	0.70
*MPL*	7/131 (5.3%)	6/123 (4.9%)	1/8 (12.5%)	0.36
*JAK2 EXON12*	0/70 (0.0%)	0/65 (0.0%)	0/5 (0.0%)	
Spleen palpable				
No	152/218 (69.7%)	136/193 (70.5%)	16/25 (64.0%)	
Yes	64/218 (29.4%)	55/193 (28.5%)	9/25 (36.0%)	0.60
Previously splenectomized	2/218 (0.9%)	2/193 (1.0%)	0/25 (0.0%)	
Size below costal margin (cm)	5.0 (2.0–7.0)	4.5 (2.0–7.0)	6.0 (4.0–20.0)	0.11
MPN-treatment before COVID				
Phlebotomy	38/230 (16.5%)	36/204 (17.6%)	2/26 (7.7%)	0.20
Cytoreduction pre-COVID	167/230 (72.6%)	144/204 (70.6%)	23/26 (88.5%)	0.054
Type:				
HU	93/231 (40.3%)	83/205 (40.5%)	10/26 (38.5%)	0.84
Anagrelide	19/231 (8.2%)	19/205 (9.3%)	0/26 (0.0%)	0.11
Interferon	5/231 (2.2%)	5/205 (2.4%)	0/26 (0.0%)	0.42
Ruxolitinib	40/231 (17.3%)	29/205 (14.1%)	11/26 (42.3%)	<0.001
Other	11/231 (4.8%)	9/205 (4.4%)	2/26 (7.7%)	0.36
Characteristics of Covid-19				
Wave				
2 (Alpha, Beta, Gamma)	14/231 (6.1%)	11/205 (5.4%)	3/26 (11.5%)	0.20
3 (Delta)	94/231 (40.7%)	79/205 (38.5%)	15/26 (57.7%)	0.061
4 (Omicron)	123/231 (53.2%)	115/205 (56.1%)	8/26 (30.8%)	0.015
COVID-19 severity^a^				
Asymptomatic infection	11/220 (5.0%)	11/197 (5.6%)	0/23 (0.0%)	0.61
Mild Illness	190/220 (86.4%)	180/197 (91.4%)	10/23 (43.5%)	<0.001
Moderate Illness	6/220 (2.7%)	4/197 (2.0%)	2/23 (8.7%)	0.12
Severe/critical Illness	13/220 (5.9%)	2/197 (1.0%)	11/23 (47.8%)	<0.001
Respiratory support	19/231 (8.2%)	3/205 (1.5%)	16/26 (61.5%)	<0.001
ICU	5/230 (2.2%)	1/204 (0.5%)	4/26 (15.4%)	<0.001
Saturation	98.0 (96.0–99.0)	98.0 (97.0–99.0)	92.0 (88.0–94.0)	<0.001
Lab values at COVID diagnosis				
Hb	12.9 (11.3–14.0)	13.0 (11.5–14.1)	11.5 (9.0–13.1)	0.002
HCT	40.0 (36.0–44.0)	40.5 (36.7–44.5)	37.5 (27.3–40.1)	0.004
RDW	16.5 (14.3–18.7)	16.0 (13.9–18.1)	18.3 (15.4–18.9)	0.093
WBC	7.5 (5.5–10.5)	7.5 (5.7–10.2)	8.1 (4.8–11.5)	0.93
Neutrophils	68.9 (56.9–76.0)	67.1 (56.3–75.0)	75.3 (63.0–82.8)	0.028
Lymphocytes	18.2 (10.9–26.8)	19.8 (12.0–28.2)	13.1 (7.4–18.5)	0.005
Neutrophils/lymphocytes ratio	3.6 (2.1–6.0)	3.3 (2.0–5.3)	5.9 (3.6–10.2)	<0.001
PLT	401.0 (226.0–608.5)	430.0 (274.0–622.0)	199.0 (119.0–294.0)	<0.001
LDH	243.0 (200.0–470.0)	236.5 (191.0–447.0)	415.0 (269.0–573.0)	0.022
CRP	8.9 (1.4–32.9)	2.0 (0.9–16.0)	33.1 (17.0–123.0)	<0.001
Fibrin	402.0 (339.0–579.0)	339.0 (309.0–480.0)	432.0 (360.0–726.0)	0.12
D-dimer	510.0 (270.0–759.0)	331.5 (185.0–568.5)	650.0 (440.0–910.0)	0.015
Vaccine information				
Months from last vaccine dose to infection	4.0 (1.4–6.6)	4.0 (1.5–6.7)	2.6 (1.2–6.0)	0.37
N° administered doses				
Only 1 dose	22/231 (9.5%)	19/205 (9.3%)	3/26 (11.5%)	0.90
2 doses	115/231 (49.8%)	103/205 (50.2%)	12/26 (46.2%)	
3–4 doses	94/231 (40.7%)	83/205 (40.5%)	11/26 (42.3%)	
Vaccine type				
Pfizer/BioNTech	179/231 (77.5%)	164/205 (80.0%)	15/26 (57.7%)	0.010
Moderna	28/231 (12.1%)	22/205 (10.7%)	6/26 (23.1%)	0.069
AstraZeneca	18/231 (7.8%)	14/205 (6.8%)	4/26 (15.4%)	0.13
Johnson&Johnson	5/231 (2.2%)	4/205 (2.0%)	1/26 (3.8%)	0.45
UNK	1/231 (0.4%)	1/205 (0.5%)	0/26 (0.0%)	–
Outcome				
Death	5/231 (2.2%)	2/205 (1.0%)	3/26 (11.5%)	0.011

^a^According to the NIH COVID-19 Treatment Guidelines.

Compared with the normal population of the 2 US cohorts of patients experiencing COVID-19 after vaccination [13], the proportion of hospitalization in our patients is markedly higher (11% vs 4.4% and 1.8%). This difference was not attributable to the number of vaccine shots in home-managed and in hospitalized patients and the two groups did not differ regarding the time between the last shot of vaccination and the onset of infection, likely excluding that hospitalization could be attributed to the waning of vaccine protection. This possibility is particularly relevant with Delta and Omicron variants which are able, at least partially, to evade vaccine-induced immunity [11].

In a multivariable logistic model fitted to predict hospitalization, we found that this risk was age-related and substantially higher in males on ruxolitinib (Fig. 1S, panel A). Interestingly, these two factors were also related to a progressive increase of NLR inflammatory biomarker (Fig. 1S, panel B), suggesting a connection between sex, aging and inflammation.

In conclusion, we have provided quantitative estimates of SARS-CoV2 infections in vaccinated patients with MPN revealing a higher rate of severe disease than in the normal population. Compared to unvaccinated, a trend for a lower reinfection rate was found in patients with hybrid vaccination. In COVID-naïve MPN patients, current original vaccine shots have shown a limited protection against delta and omicron VOCs. While waiting for the results of the new boosters targeting the newest strains of the omicron variants, we suggest a prompt identification of the MPN subgroup at high risk of hospitalization, in which early treatment with the recent monoclonal antibodies against the spike protein of the SARS-CoV-2 virus, anti-inflammatory and antiviral drugs can be suggested.

## Supplementary information

Figure 1S

## Data Availability

Aggregated data available by request. Patient-level data will not be shared.
