# Supplementary material for: Breakthrough infections in MPN-COVID vaccinated patients
Source: Blood Cancer J. 2022 Nov 15;12(11):154. doi: 10.1038/s41408-022-00749-8 (PMC9664028; doi:10.1038/s41408-022-00749-8)

**SUPPLEMENTARY MATERIAL**

**Figure 1S. Probability of hospitalization according to (A) age, ruxolitinib exposure and gender and (B) neutrophils/lymphocytes ratio, ruxolitinib exposure and gender**


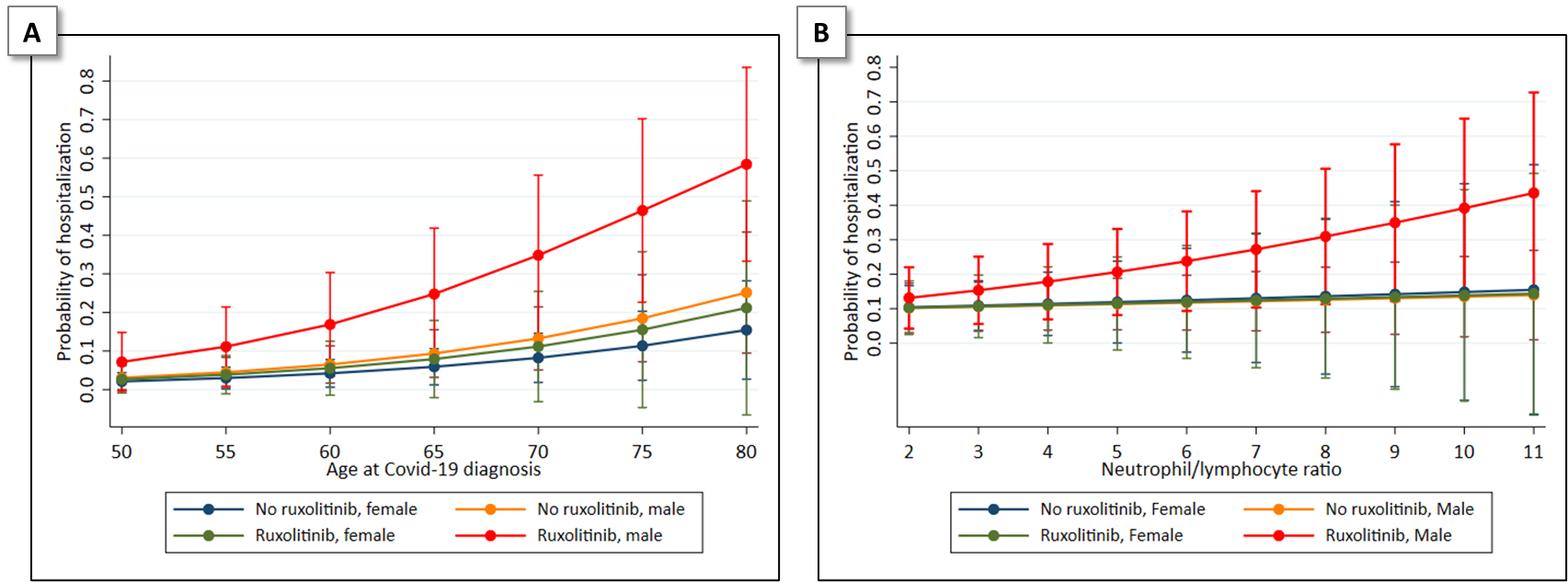

Supplement: Supplementary file 1 — Figure 1S [file 41408_2022_749_MOESM1_ESM.docx]
